# Systemic therapy and radiotherapy related complications and subsequent hospitalisation rates: a systematic review

**DOI:** 10.1186/s12885-024-12560-8

**Published:** 2024-07-10

**Authors:** Rashidul Alam Mahumud, Md. Shahjalal, Padam Kanta Dahal, Md. Parvez Mosharaf, Mohammad Enamul Hoque, Olivia Wawryk

**Affiliations:** 1 NHMRC Clinical Trials Centre, Faculty of Medicine and Health, The University of Sydney, Camperdown, New South Wales (NSW) Australia; 2 Global Health Institute, North South University, Dhaka, Bangladesh; 3 School of Health, Medical and Applied Sciences, Central Queensland University, Sydney Campus, Sydney, Australia; 4 School of Business, Faculty of Business, Education, Law and Arts, University of Southern Queensland, Toowoomba, QLD Australia; 5 Faculty of Medicine and Health, The University of Western Australia, Perth, Australia; 6 Department of General Practice, Victorian Comprehensive Cancer Centre, Data Connect, University of Melbourne, Parkville, VIC Australia

**Keywords:** Systemic therapy, Radiotherapy, Hospitalisation, Length of stay, Complications, Cancer patients

## Abstract

**Background:**

Hospitalisation  resulting from complications of systemic therapy and radiotherapy places a substantial burden on the patient, society, and healthcare system. To formulate preventive strategies and enhance patient care, it is crucial to understand the connection between complications and the need for subsequent hospitalisation. This review aimed to assess the existing literature on complications related to systemic and radiotherapy treatments for cancer, and their impact on hospitalisation rates.

**Methods:**

Data was obtained via electronic searches of the PubMed, Scopus, Embase and Google Scholar online databases to select relevant peer-reviewed papers for studies published between January 1, 2000, and August 30, 2023. We searched for a combination of keywords in electronic databases and used a standard form to extract data from each article. The initial specific interest was to categorise the articles based on the aspects explored, especially complications due to systemic and radiotherapy and their impact on hospitalisation. The second interest was to examine the methodological quality of studies to accommodate the inherent heterogeneity. The study protocol was registered with PROSPERO (CRD42023462532).

**Findings:**

Of 3289 potential articles 25 were selected for inclusion with ~ 34 million patients. Among the selected articles 21 were cohort studies, three were randomised control trials (RCTs) and one study was cross-sectional design. Out of the 25 studies, 6 studies reported ≥ 10 complications, while 7 studies reported complications ranging from 6 to 10. Three studies reported on a single complication, 5 studies reported at least two complications but fewer than six, and 3 studies reported higher numbers of complications (≥ 15) compared with other selected studies. Among the reported complications, neutropenia, cardiac complications, vomiting, fever, and kidney/renal injury were the top-most. The severity of post-therapy complications varied depending on the type of therapy. Studies indicated that patients treated with combination therapy had a higher number of post-therapy complications across the selected studies. Twenty studies (80%) reported the overall rate of hospitalisation among patients. Seven studies revealed a hospitalisation rate of over 50% among cancer patients who had at least one complication. Furthermore, two studies reported a high hospitalisation rate (> 90%) attributed to therapy-repeated complications.

**Conclusion:**

The burden of post-therapy complications is emerging across treatment modalities. Combination therapy is particularly associated with a higher number of post-therapy complications. Ongoing research and treatment strategies are imperative for mitigating the complications of cancer therapies and treatment procedures. Concurrently, healthcare reforms and enhancement are essential to address the elevated hospitalisation rates resulting from treatment-related complications in cancer patients.

**Supplementary Information:**

The online version contains supplementary material available at 10.1186/s12885-024-12560-8.

## Introduction

Recent advances in cancer treatment [1], including systemic therapy and radiotherapy, have redefined the landscape of oncological care [2–4]. These treatment modalities, individually and in combination, have significantly improved patient outcomes and survival rates [5, 6]. While systemic therapy and radiotherapy have improved overall survival rates, they have also been linked to adverse events, such as neutropenia, thrombocytopenia, anaemia, sepsis, fever, mucositis, dehydration, and nausea and vomiting, some of which can result in hospitalisations [7–10]. There may be a complex relationship of treatment-related complications that can give rise to scenarios necessitating hospitalisation. This complex relationship between systemic therapy, radiotherapy, and subsequent hospitalisation has a profound impact on comprehensive cancer care [9].

A number of studies have examined the effectiveness of these treatments in terms of disease control and patient survivorship [11–14]. However, the impact of treatment-related complications and their correlative effects on hospitalisation rates have yet to be synthesised. This review seeks to address this knowledge gap, shedding light on the multifaceted relationship that shapes the trajectory of cancer care.

Systemic therapy includes a range of drug treatments or interventions, including chemotherapy, immunotherapy, and targeted therapy, each precisely tailored to the unique attributes of individual patients, the nature of their diseases, and the predominant treatment objectives [11, 13, 15–18]. Several complications occurring from systemic therapy and radiotherapy cover a diverse range [7–10], involving both immediate and long-term effects which have serious repercussions on the quality of life and survival outcomes [5, 19–21]. These complications are evidenced as haematological toxicities, neurological impairments, gastrointestinal perturbations, dermatological problems, and cardiopulmonary sequelae, among various others [9, 22, 23]. While certain complications can be effectively managed through outpatient healthcare services, others refer to a more significant impact, necessitating patients to hospitalisation for meticulous observation, intensive monitoring and specialised interventions.

The evolution of cancer care has gone beyond the boundaries of the clinic, reflecting shifts in outpatient-centred care models, and augmented supportive care strategies. Reducing hospitalisations has been a significant improvement in patient experience, meeting the patient's desire to maintain their health status. However, hospitalisation due to complications of systemic therapy and radiotherapy can have a significant burden on the patient, society, and the healthcare system. These hospitalisations can lead to interruptions in treatment and adversely affect the patient's response to treatment. Furthermore, complications resulting in hospitalisations can also result in treatment interruptions and negatively affect the patient's response to treatment. Although some review studies have attempted to capture post-therapy complications due to systemic therapy and/or radiotherapy, most of these studies have focused on a single type of cancer or a specific type of complication [24–29]. Unfortunately, there has been little effort to conduct a comprehensive review study that provides extensive data on all types of complications, any type of cancer, various types of therapy, and their subsequent hospitalisation.

The increasing concern about the burden of hospitalisation due to treatment-related complications has made it crucial to understand the link between complications and hospitalisation. This knowledge is essential for the development of preventive strategies and the improvement of patient care. Therefore, the primary objective of this systematic review is to analyse the available ongoing literature to investigate the relationship between systemic therapy and radiotherapy-related complications, and how they affect hospitalisation rates. This review presents a comprehensive analysis of the existing literature on complications related to systemic and radiotherapy treatments for cancer, and their impact on hospitalisation rates. The review highlights that hospitalisations due to treatment complications pose a significant burden on the healthcare system. When many patients require hospitalisation due to complications, it can reduce the availability of resources for other patients in need, limiting access to care. Moreover, increased hospitalisations can lead to additional demands on healthcare professionals, potentially increasing their workload and affecting patient care quality. Policymakers and clinicians can use this evidence to revisit existing clinical policies, regulations, and strategies, while patients can make better-informed decisions regarding their cancer therapies. This review can serve as a guide for future intervention efforts and contribute to a better scientific understanding of the issue.

By analysing a diverse range of studies, the objective of this review is to achieve the following goals:

1. Identify the types and frequencies of complications associated with systemic therapy and radiotherapy.
2. Quantify the proportion of patients who require hospitalisation due to complications arising from these treatments.
3. Investigate potential predictors of hospitalisation, such as patient demographics, treatment modalities, and complication severity.
4. Discuss the implications of treatment-related complications for healthcare resource allocation, patient well-being, and treatment decision-making.

## Methods

This systematic review was registered under International Prospective Register of Systematic Reviews (PROSPERO) with the registration number of CRD42023462532. This review adopted the PICOS framework for structing and designing this study [30].

**Table:** 

**PICOS**	**Population (P):** All patients undergoing systemic therapy and/or radiotherapy for cancer treatment
	**Interventions (I):** Systemic therapy and/or radiotherapy for cancer treatment
	**Comparison (C):** No specific comparison is required for this systematic review since the focus is on the relationship between treatment complications and subsequent hospitalisation rates
	**Outcomes (O):** o Types and frequencies of complications associated with systemic therapy and radiotherapy o Proportion of patients experiencing complications that lead to hospitalisation o Variation in hospitalisation rates across different cancer types and treatment contexts o Implications of treatment-related complications for healthcare resource allocation, patient well-being, and treatment decision-making
	**Study Design (S):** This review comprehensively analyses the impact of systemic therapy and radiotherapy complications on hospitalisation rates, using various study designs including randomized controlled trials, cohort studies, and cross-sectional observational studies

### Eligibility criteria

This review added a published peer-review publication provided it fulfilled all the following criteria. In this review, inclusion and exclusion criteria were defined based on the research objectives, study population, interventions, comparisons, and outcomes (PICOS framework) [31]. An eligible article was selected based on the following criteria:

### Inclusion criteria

1. All patients underwent systemic therapy and/or radiotherapy for cancer treatment.
2. Original research articles using quantitative study designs (e.g., cohort, longitudinal, case–control, cross-sectional, and randomised controlled trials),
3. Studies reported complications or side effects associated with systemic therapy and/or radiotherapy.
4. Studies stated the proportion of patients experiencing complications that require hospitalisation.
5. Studies that provided sufficient data for the synthesis of outcomes.
6. Studies published in English and published between January 1, 2000 to August 30, 2023.

### Exclusion criteria

This review excluded these publications, including reviews, perspectives, opinion speech of papers, commentaries, editorials, letters, conference abstracts, reports, grey literature, unpublished research, studies without primary data, animal studies, laboratory studies, and in vitro studies.

### Information sources

A search strategy was developed to search Medline via the PubMed interface, Scopus, Embase, and Google Scholar online databases to identify relevant original peer-reviewed papers for our systematic review. In addition to the database search, we explored references of selected studies and previously published article on similar topics (backward and/or forward reference searching) to incorporate all potential pertinent articles to construct our summary estimates. A snowball method was applied to ensure completeness.

### Search strategy

This systematic review was conducted to identify literature that reported on complications due to systemic and radiotherapy and their impact on hospitalisation. The search strategy was comprised of three concepts: (i) systemic therapy and radiotherapy as part of cancer treatment; (ii) complications or side effects associated with systemic therapy and/or radiotherapy; (iii) hospitalisation due to therapy-related complications. This review screened quantitative studies focused on cancer diseases. This review search included articles published in English language between January 1, 2000, and August 30, 2023, to capture the most relevant and update articles on this topic. A combination of keywords, MeSH and Boolean operators’ terms were applied to develop the search terms. A description of search terms is given in Appendix Table A1.

### Data collection process

The four reviewers (RAM, MS, PKD, and MPM) screened the titles and abstracts of retrieved studies against the predefined inclusion and exclusion criteria. After settling any differences, the reviewers independently extracted the data, discussed the inputs, and revised the extracted data. Unresolved issues were resolved by involving two reviewers (OW and MEH). Excluded studies did not meet the eligibility criteria.

### Data items

This review considered the following outcomes according to the study objectives:

- Types and frequencies of complications associated with systemic therapy and radiotherapy.
- Proportion of patients experiencing complications that lead to hospitalisation.
- Variation in hospitalisation rates across different cancer types and treatment contexts.

### Data extractions

Using EndNote libraries, four independent reviewers screened the articles. They created a data-extraction form to establish the type of information to be extracted. The reviewers recorded relevant data on the name of the first author, study settings (country), publication year, study design, number of study participants, time horizon /follow-up periods, demographics (e.g., average age/age group, gender), cancer types, cancer stages, systemic and radiotherapy, intervention, comparators, list of complications and associated hospitalisations. We further inputted data on hospitalisations (length of stay in days, and/or percentage of hospitalised patients). In addition, they documented studies’ description of analytical models/methods, internal validity checks or robustness of findings, description of handling missing data, and funding sources. Unresolved issues were resolved by involving a reviewer (OW).

### Quality assessment

Considering the diverse range of study designs including randomised controlled trials (RCTs) [32], cohort, and cross-sectional studies [33], three distinct quality assessment tools were employed to accommodate the inherent heterogeneity. Reviewers assessed the quality of the included studies, and the discrepancies were resolved with discussion with the reviewer (OW). The critical appraised tools utilised in this study were constructed by the Joanna Briggs Institute (JBI) [33]. These tools are widely adopted in academic studies [34, 35] and provide a nuanced evaluation of the risk of bias, categorised as low, moderate, or high [35]. Study quality impacts future research confidence. High-quality studies assure conclusions, while low-quality studies raise replication doubts.

### Synthesis of results

The reviewers used a narrative synthesis approach for synthesis of results that integrated the quantitative findings with the qualitative insights. This approach facilitated a comprehensive understanding of the relationships between systemic therapy, radiotherapy, complications, and hospitalisation rates. Using this approach, the reviewers observed the patterns, trends, and consistencies across studies within this qualitative synthesis. The reviewers used Microsoft Excel to manage synthesis data and analyse data. To present the relationship between post-therapy complications and the reporting studies, a heatmap was generated using the statistical software R (v4.3.1).

### Synthesis methods

Data were systematically presented and tabulated to encompass population demographics and study characteristics. Within the scope of descriptive analysis, categorical variables' attributes were interpreted through frequencies (*n*) and percentages (%), which were employed to convey the continuous nature of quantitative data. The comprehensive account of systemic and radiotherapy-related complications was outlined, categorised by cancer types, stages, and study designs. Pertaining to hospitalisation, data stemming from complications due to systemic and radiotherapy were demonstrated in terms of descriptive statistics, including mean length of stay (in days) or hospitalisation rates expressed as a percentage, as appropriate to the context.

## Results

### Data selection and process

**Fig. 1:**
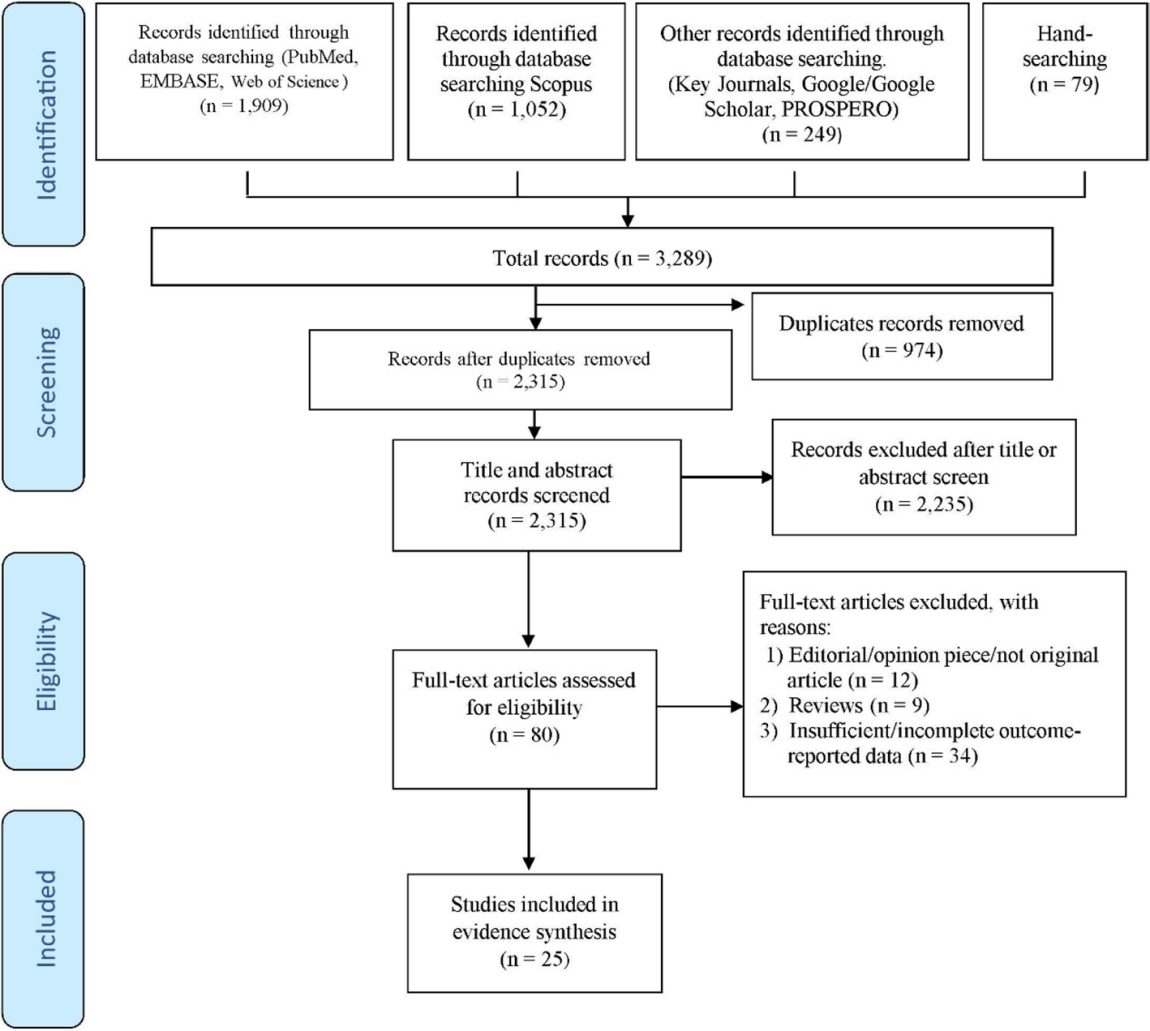
PRISMA flow diagram

The eligibility of studies included was determined following a three-steps screening process. Firstly, EndNote software was used to eliminate duplicates from all retrieved studies or documents. Secondly, the reviewers examined the articles by reading the titles and abstracts to determine their relevance to our study. Finally, the third stage necessitated reading of full texts of all possibly relevant articles identified by our searches as reflected in the PRISMA flow diagram Fig. 1.

### Description of studies included

The initial search retrieved 3289 studies from the electronic databases. After excluding the duplicates and irrelevance or absence of required information, we screened the titles and abstracts for a further selection of eligible articles (Fig. 1). Subsequently, we selected 80 articles based on eligibility criteria for full-text review. After excluding 55 studies in the full-text review, finally, 25 studies with a total of approximately 33.82 million patients were included in the systematic review [7–10, 15, 18, 21, 36–53]. A total of 55 studies were excluded for various reasons: 12 were editorial, opinion pieces, commentary, or letters to the editor; 9 were review papers; and 34 did not provide sufficient or complete outcome-related data.

### Characteristics of the studies

Table 1 shows the characteristics of the included studies in our review. Approximately 52% studies (12 out 25 studies) were in the USA (*n* = 13) [7, 9, 10, 15, 18, 37, 38, 40, 45, 46, 49, 51, 53], followed by 28% studies in Australia (*n* = 7) [8, 21, 36, 39, 41, 42, 44]. In terms of study participants, the selected studies exhibited a wide range, with participant numbers varying from 60 to 29,546,02 participants. 56% of the studies (14 out of 25 studies) involved 1000 or more participants [9, 10, 15, 18, 37, 38, 40, 42, 44–46, 48, 51, 53], while approximately one-third of the selected studies (7 out of 25 studies) had participants number ranging from 100 to 500 [7, 8, 21, 36, 41, 50, 52]. One study had a comparatively smaller participants number (< 100 participants) compared with others [39]. Furthermore, most selected studies (21 out of 25 studies) adopted a cohort study design [7–10, 21, 36–39, 41–46, 48, 49, 51–54], three RCTs [47, 49, 52] and one study using a cross-sectional study design [40].

**Table 1:**
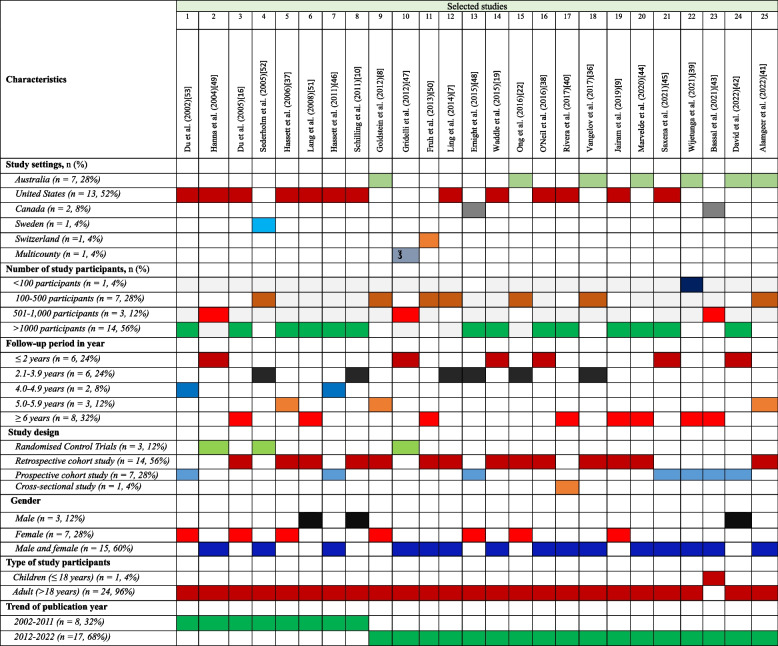
Study background characteristics

^€^16 countries (Australia, Belgium, Canada, Finland, France, Germany, Greece, India, Italy, The Netherlands, Poland, Portugal, Romania, Spain, Turkey, and the United Kingdom)

A higher number of selected studies (60%, 15 out of 25) included both male and female populations. Seven studies (28%) exclusively focused on female participants, while three studies included male participants only (Table 1). Most of the selected studies (96%, 24 out of 25) featured adult (> 18 years) participants, while one study solely focused on child participants [43]. Considering the trend of relevant publications, eight of the selected studies (32%) were published between 2002 and 2011, while 17 studies (68%) were published between 2012 and 2022 (Table 1).

### Cancer and diagnosis related information

Table 2 shows the participants' cancer-related characteristics. Out of the 25 studies reviewed, 15 of them focused on patients with a single type of cancer (breast, lung or other) [8, 15, 21, 36, 37, 39, 42, 43, 47–53], while the remaining 10 studies included individuals with multiple types of cancers [7, 9, 10, 38, 40, 41, 44–46]. Furthermore, five studies were conducted solely on breast cancer patients, and six focused on lung cancer patients. Approximately 62% of the studies (15 out of 25) reported on patients with cancer at all stages, from stage I to IV. Nine studies focused on patients with advanced-stage cancer (stage III or IV), while one study reported early-stage cancer (stage I or II). In terms of the treatment modalities used in the studies, 9 out of 25 studies (55%) employed a combination of therapies, such as chemotherapy, radiotherapy, hormonal therapy, and/or antibiotic therapy for cancer treatment. On the other hand, 16 out of 25 studies reported that patients received a single therapy, either chemotherapy or radiotherapy. The details are presented in Table 2.

**Table 2:** Distribution of cancer types, stages and therapies

Study	Types of cancer			Cancer stage		Cancer-related therapy	Intervention		Comparator	
Du et al. [53]	Breast cancer			all		Chemotherapy	Drug treatment		NR	
Hanna et al. [49]	Non–small-cell lung cancer			III/IV		Chemotherapy	Drug treatment		Existing standard care	
Du et al. [15]	Breast cancer			all		Chemotherapy	Drug treatment		Modes of chemotherapy administration	
Sederholm et al. [52]	Non–Small-Cell Lung Cancer			III/IV		Chemotherapy and Radiotherapy	Drug treatment and Radiotherapy		NR	
Hassett et al. [37]	Breast cancer			all		Chemotherapy, Radiation therapy, Hormonal therapy	Surgery with radiotherapy		Radiation therapy	
Lang et al. [51]	Lung cancer			III/IV		Chemotherapy	Drug treatment		NR	
Hassett et al. [46]	Breast, colorectal, and lung			all		Chemotherapy	Drug treatment		Chemotherapy receiver vs not receiver	
Schilling et al. [10]	Hematologic, lung or bronchial, breast			III/IV		Chemotherapy, Antibiotic therapy	Drug treatment		Antibiotic therapy	
Goldstein et al. [8]	Breast cancer			III		Chemotherapy, Chemoradiation therapy	Surgery Ivor-Lewis, Thoracoscopic 3 stage		Chemotherapy & Surgery vs Chemoradiation therapy & Surgery, No surgery	
Gridelli et al. [47]	Lung cancer			III/IV		Chemotherapy	Drug treatment		Placebo	
Fruh et al. [50]	Lung cancer			IV		Chemotherapy	Drug treatment		NR	
Ling et al. [7]	Head and neck cancer			all		Chemotherapy and Radiotherapy	Radiotherapy plus radiotherapy		Existing standard care	
Ernight et al. [48]	Breast cancer			I-III		Chemotherapy	Drug treatment		Receiver vs not receiver	
Waddle et al. [18]	Multiple cancers^c^			all		Radiotherapy	Radiotherapy		NR	
Ong et al. [21]	Testicular cancer			I/II		Radiotherapy	Surgery with radiotherapy		NR	
O'Neil et al. [38]	Multiple cancers^b^			all		Chemotherapy	Drug treatment		No Chemotherapy	
Rivera et al. [40]	Multiple cancers^a^			all		Chemotherapy or Radiotherapy	Chemotherapy or Radiotherapy		NR	
Vangelov et al. [36]	Oropharynx cancer			III/IV		Radiotherapy, Chemotherapy	Surgical intervention		Prophylactic feeding tube vs reactive feeding tube	
Jairam et al. [9]	Multiple cancers			III/IV		Systemic therapy and Radiotherapy	Systemic therapy and Radiotherapy		NR	
Marvelde et al. [44]	Solid and Haematological cancers			all		Chemotherapy and Radiotherapy	Chemotherapy or Radiotherapy		NR	
Wijetunga et al. [39]	Pancreatic cancer			all		Neoadjuvant chemotherapy	Drug treatment		NR	
Bassal et al. [43]	Acute lymphoblastic leukemia			all		Chemotherapy	Drug treatment		Steroids (dexamethasone vs. prednisone) and versions of interim maintenance (Capizzi vs. high-dose methotrexate)	
Saxena et al. [45]	Multiple cancers^d^			all		Systemic therapy	Drug treatment		NR	
David et al. [42]	Prostate cancer			all		Radiotherapy	Radiotherapy		NR	
Alamgeer et al. [41]	Head & neck squamous cell carcinoma			all		Chemotherapy and Radiotherapy	Chemotherapy or Radiotherapy		Standard vs non-standard regiment	

*NR *Not reported, *all *all type of stages

^a^Breast, prostate, lung, and multiple cancers, including secondary malignancy, multiple myeloma, pancreatic cancer, rectal cancer

^b^Breast, colorectal, ovarian, bladder, lung, pancreas, esophageal, stomach, or prostate cancer, small-cell lung

^c^Bone metastasis, brain metastasis, genitourinary; gynaecological; haematological, skin, and bone and soft tissue malignancies

^d^Multiple cancers (bladder, bone and connective tissue, brain and nervous system, breast, cervix, colon, esophagus, head and neck, kidney and renal, liver and intrahepatic bile duct, lung, melanoma, other skin cancers, ovary, pancreas, prostate, rectum and anus, stomach, testis, thyroid, uterus, hodgkin lymphoma, non-hodgkin lymphoma, multiple myeloma and leukemia)

### Reported therapy-related complication and impact on hospitalisation

Table 3 presents data on complications related to therapy and resulting hospitalisations. Most studies (19 out 25 studies) reported multiple complications, but three studies focused on a single complication [21, 36, 39]. Out of the 25 studies, six studies reported 10 or more complications [7–10, 37, 47], while seven studies reported complications ranging from six to ten (Table 3). Five studies reported at least two complications but fewer than six [38, 42, 43, 50, 51], and three studies reported significantly higher numbers of complications (15 or more) compared with other selected studies. Furthermore, four studies reported between nine to eleven complications. However, three out 25 studies concentrated on a single complication [21, 36, 39], including weight loss, fatigue, and reduced lymphovascular invasion.

**Table 3:** Reported therapy-related complications and hospitalisation

Study	Reported number complications	Hospitalisation
Du et al. [53]	Neutropenia, fever, and thrombocytopenia (*n* = 3)	Overall = 9%
Hanna et al. [49]	Neutropenia, febrile neutropenia, neutropenia with infection, anemia, and thrombocytopenia (*n* = 5)	Overall = 17%
Du et al. [15]	Neutropenia, thrombocytopenia, fever, infection, dehydration, and delirium (*n* = 6)	Overall = 54.37%
Sederholm et al. [52]	Anemia, leucopenia, thrombocytopenia, alkaline phosphatase, alopecia, Aspartate aminotransferase (AST)/ Alanine aminotransferase (ALT), bilirubin, cardiac function, constipation, diarrhea, hematuria, infection, nausea/vomiting, pulmonary, serum creatinine, state of consciousness, and bleeding episodes (*n* = 17)	Overall = 25%
Hassett et al. [37]	Infection, fever, neutropenia, thrombocytopenia, anaemia, nausea, emesis, diarrhea, malnutrition, constitutional, nonspecific symptoms, dehydration, electrolyte disorders, deep venous thrombosis, or pulmonary embolus (*n* = 15)	Overall = 51% (ALOS = 5 days)
Lang et al. [51]	Anemia, dehydration, infection, and neutropenia (*n* = 4)	Overall = 46%
Hassett et al. [46]	Cardiac, gastrointestinal, heanmtologic, and infectious (*n* = 4)	Overall = 8.7%
Schilling et al. [10]	Neutropenia, neutropenia plus neutropenia infection or fever, neutropenia without infection of fever, neutropenia, and infection (*n* = 10)	ALOS = 9 days
Goldstein et al. [8]	Oesophagitis, pneumonitis, neutropenia, skin, mucositis, fever, infection, nausea/vomiting, thromboembolism, renal impairment, chest problems, cardiac problems, anastomotic leak, wound infection, and chyle leak (*n* = 16)	ALOS = 12 days
Gridelli et al. [47]	Anemia, neutropenia, fatigue, anorexia, constipation, diarrhea, muscositis, nausa, vomitting, edema, neuropathy, and eye pain (*n* = 12)	Overall = 29%
Fruh et al. [50]	Hematoxcity, and neurotoxicity (*n* = 2)	Overall = 40%
Ling et al. [7]	Coronary artery disease, myocardial infarction; peripheral vascular disease; history of deep vein thrombosis; diabetes; hypertension; and chronic kidney disease dehydration, dysphagia, odynophagia, mucositis, nausea, vomiting, neutropenic fever, and syncope (*n* = 15)	Overall = 25.2%
Ernight et al. [48]	Infection, and gastrointestinal causes (*n* = 2)	Overall = 6.9%
Waddle et al. [18]	Vomiting, diarrhea, altered mental status or delirium, pneumonia or shortness of breath, pain, fever, or infection (*n* = 8)	Overall = 20%
Ong et al. [21]	Fatigue (*n* = 1)	Overall = 25%
O'Neil et al. [38]	Gastrointestinal complications, malnutrition, cardiac complications, or constitutional symptoms (*n* = 4)	Overall = 92%
Rivera et al. [40]	Aspiration pneumonitis, renal failure, fracture of neck of femur (hip), pulmonary heart disease, acute myocardial infarction, intestinal infection, respiratory failure, acute cerebrovascular disease, and pathological fracture (*n* = 9)	Overall = 65%
Vangelov et al. [36]	Weight loss (*n* = 1)	ALOS = 9 days
Jairam et al. [9]	Neutropenia, dehydration, radiation enteritis sepsis, nausea and vomiting, disorders of rectum and anus anaemia, acute kidney injury, intestinal obstruction, pneumonia, and radiation cystitis (*n* = 11)	Overall = 90.9%
Marvelde et al. [44]	Respiratory, cardiovascular, renal, hepatic, haematologic, metabolic, and neurological (*n* = 7)	ALOS = 9 days
Saxena et al. [45]	Anemia, neutropenia, sepsis, pneumonia, acute kidney injury, nausea with vomiting, dehydration, urinary tract infection, congestive heart failure, and fever of unknown origin (*n* = 10)	Overall = 12% (ALOS = 4.5 days)
Wijetunga et al. [39]	Reduced lymphovascular invasion (*n* = 1)	Overall = 58.3%
Bassal et al. [43]	Infection, and neutropenia (*n* = 2)	Overall = 76.2%
David et al. [42]	Haematuria, irradiation cystitis, urethral stricture, urinary incontinence, and urinary retention (*n* = 5)	Overall = 20%
Alamgeer et al. [41]	Mucositis, dysphagia, malnutrition, infection & sepsis, febrile neutropenia, nausea, and vomiting (*n* = 9)	Overall = 33.6%

**Fig. 2:**
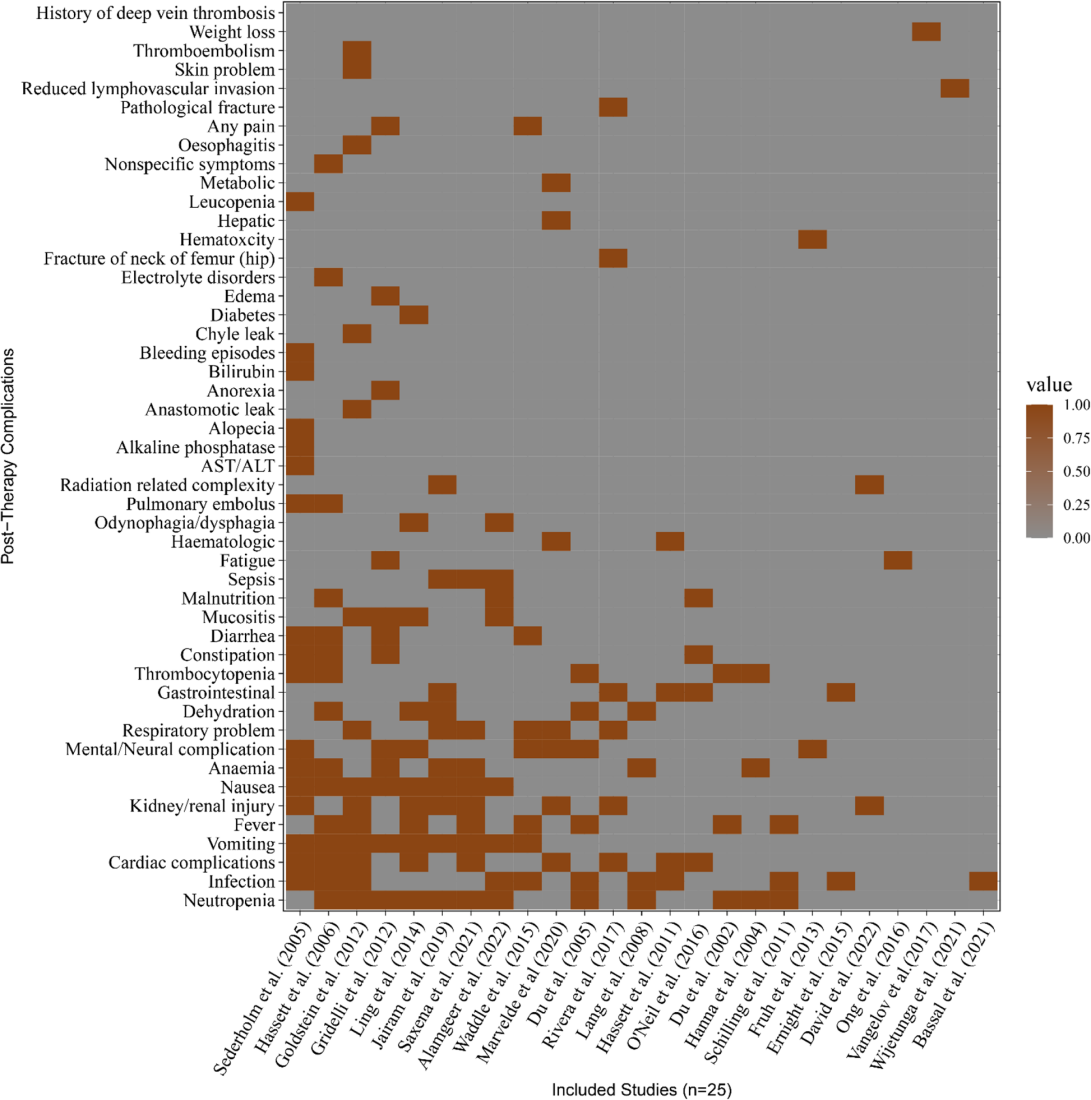
The distribution and relationship between the post-therapy complications and the selected studies. Note: The complications were plotted in the y-axis corresponding to the included studies in the x-axis. The orange boxes mean the presence (value = 1) of that type of complication reported by the corresponding studies and the grey boxes mean the absence (value = 0) of that type of complication. AST = aspartate aminotransferase and ALT = alanine aminotransferase

In this study, we investigated the reported post-therapy complications to generalize the disease patterns. Compiling the adverse events, we have classified and found 49 different complications based on reported complication types and characteristics (Supplementary File S1). Among the reported complications, neutropenia, any types of infections, cardiac complications, vomiting, fever, and kidney/renal injury were the top-most post-therapy complications (Fig. 2). Moreover, anemia, mental/neural complications, and respiratory complications were also reported by the included studies as post-therapy complications.

**Fig. 3:**
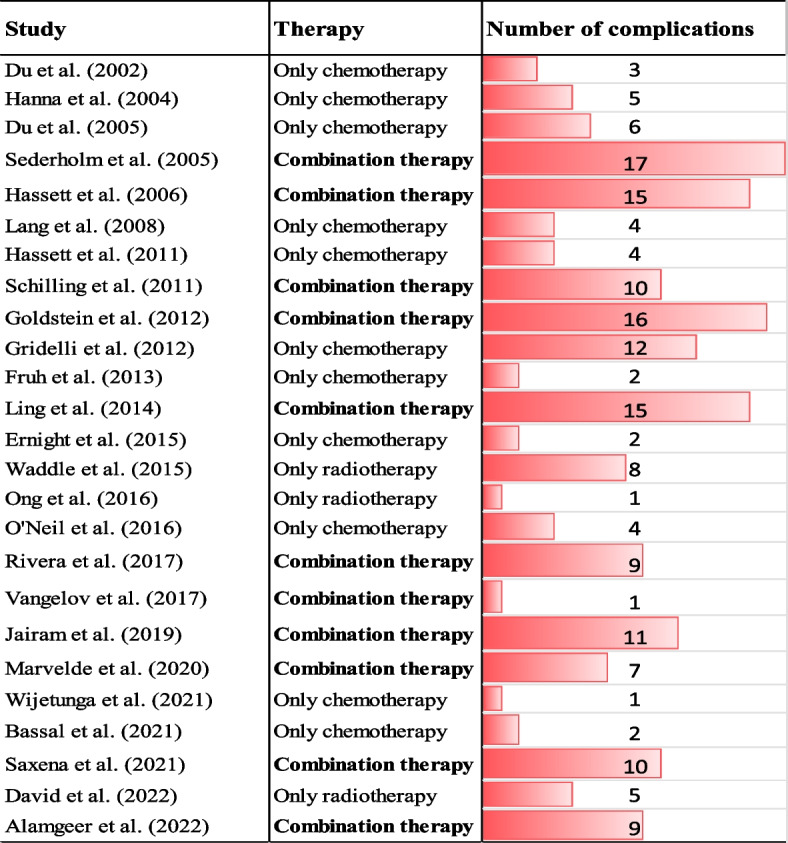
Number of post-therapy complications by therapy. Note: Combination therapy involves using two or more treatment methods simultaneously. This could include chemotherapy and radiotherapy; chemotherapy, radiation therapy, and hormonal therapy; or chemotherapy and antibiotic therapy. On the other hand, single therapy refers to using only one method of treatment modality, such as chemotherapy, radiotherapy, or hormonal therapy

Figure 3 shows that the severity of post-therapy complications varied depending on the type of therapy. For example, studies indicate that patients treated with combination therapy had a higher number of post-therapy complications across the selected studies. This suggests that combination therapy is associated with a higher number of post-therapy complications.

In the investigation of hospitalisations resulting from therapy-related complications, 20 studies (80%) reported the overall rate of hospitalisation among patients [7, 9, 15, 18, 21, 38–43, 45–53]. Of these, four studies provided data on the average length of stay (ALOS) [8, 10, 36, 44], while two studies reported both the overall hospitalisation rate and ALOS [37, 45]. Of the studies which reported ALOS, three out six studies showed a hospitalisation ALOS of 9 days, [10, 36, 44] whereas one study was reported an ALOS of 12 days [8]. Seven out of 25 studies revealed a hospitalisation rate of over 50% among cancer patients who had at least one complication [9, 15, 37–40, 43]. Furthermore, two studies reported an exceptionally high hospitalisation rate (> 90%) attributed to therapy-repeated complications [9, 38].

### Quality of included studies

Based on the review, it was found that all 21 cohort studies analysed were deemed reliable due to their high-quality standards [7–10, 21, 36–39, 41–46, 48, 49, 51–54]. Furthermore, three RCTs [47, 49, 52] and single cross-sectional study were assessed to be of high quality, indicating that they adhered to the JBI quality assessment criteria [40]. It's worth noting that no studies were excluded based on poor quality assessment scores (more details Appendix Table A2-A4).

## Discussion

This study analysed data from 25 studies that involved approximately 33.8 million cancer patients, providing a summarised relationship between systemic therapy and radiotherapy complications and their impact on hospitalisation rates. This review identified all studies were conducted in high-income countries such as the United States, Australia, and Canada. However, there is a lack of evidence on cancer therapy-related post-complication complications and associated hospitalisations, specifically in low- and lower-middle-income countries. The study revealed that approximately half of the included studies used combination therapies, such as chemotherapy and radiotherapy and/or hormonal therapy or antibiotic therapy) as the prevailing treatment modalities among cancer patients. This study found that a combination therapy is associated with a higher complication rate. Regarding post-therapy complications due to systemic therapy and radiotherapy, two-thirds of the included studies’ participants reported multiple complications related to therapy, with most studies reporting that around 80% of hospitalisations were associated with these complications. These findings varied depending on the type of cancer diagnosis, disease severity, and study design. For instance, among the selected studies conducted among breast or lung cancer patients, three or more complications were reported. Most of the selected studies adopted a cohort study design to generate this evidence, followed by randomised control trials. Our review identified that the majority of the cancer patients were from all stages (i.e., I to IV), however, minority studies included the advanced (III/IV) and early stages (I/II) of patients. A previous systematic review, focused on endometrial cancer, reported that severe adverse effects in the advanced stages were common [55]. Further, another study illustrated that chemotherapy and radiotherapy for all types of cancers induced changes which may present complications, leading to hospitalisations of the patients [16]. As such, cancers at advanced stages are vulnerable to treatment-related complications that could lead to hospitalisations. These emphasise the critical need for early diagnosis and tailored therapeutic approach especially in the advanced stages, including proactive management of treatment related adverse effects to reduce the rate of hospitalisation.

Similar to the studies conducted in previous [11, 13, 20], our review found that more than 50% of treatment modalities were a combination of chemotherapy and radiotherapy for cancer treatment which might lead to complications [56, 57]. We found that a combination therapy is associated with a higher complication rate. Using multiple therapeutic methods at the same time can lead to an increase in adverse events and complications. A study on advanced pancreatic ulcers highlighted that though combination treatment strategies increased the side effects, had the potential to improve the treatment outcomes [57]. This highlights the need to carefully weigh the potential benefits and risks of combining different treatments. Although combination therapy can have promising outcomes, the higher complication rate underscores the importance of considering the risk–benefit ratio when making clinical decisions. Future research should focus on understanding the mechanisms behind these complications to refine treatment protocols, improve patient safety, and ultimately enhance the effectiveness of combination therapies in managing this specific condition.

Our review reports the data on the therapy-related adverse effects and patient hospitalisation rates as a major outcome, illustrating multiple complications that might lead to higher hospitalisations rate and longer hospital stay. Some previous studies reported that therapy-related treatment modalities for cancer have higher complications, these lead to higher emergency visits and length of hospital stay [9, 58, 59]. These might be due to the toxicity of treatment, weak immune system, and patient with other co-morbidities. In addition, sepsis, pneumonia and aspiration pneumonitis were associated with longer hospital stays [9, 45]. These results underscore the needs of identification and management of potential side effects related to cancer therapies. The findings also suggest the importance of a multidisciplinary approach to cancer care, involving oncologists, nurses, or other health professionals to minimise the adverse events and reduce hospitalisation rates. Therefore, the authors suggest future clinical research on refining the treatment modalities.

This review has some limitations. Firstly, most of the included studies are from developed countries, which could limit the generalisability of our findings to the low- and lower-middle-income countries, where healthcare systems and patient populations may differ substantially. Secondly, there is a paucity of uniformity in the research methodologies applied across studies. However, the majority are cohort studies, and one cross-sectional study design and this variation can affect the level of casual inferences that can be drawn from the findings. Thirdly, this review revealed variations in the follow-up periods used in the included studies. This variability can impact decision-making, potentially leading to confusion among audience regarding the long-term or short-term impacts of different cancer treatment modalities. Lastly, the availability and consistency of data regarding specific complications and hospitalisations are limited. This inconsistency poses challenges for making direct comparisons and assessing the nature and severity of complications. Despite these acknowledged and essential limitations, one of the study's strengths was its strong study selection and screening protocols. Because of our rigorous search approach and inclusion criteria, this review conducted the systematic review of hospitalisation due to therapy-related complications among cancer patients to date. Most of the papers included in the review were of high quality. The comprehensive subgroup analyses demonstrate that our findings are applicable to a wide range of contexts. The review findings underscore the importance of delving deeper into the intricate relationships between systemic and radiotherapy-related complications and their causal links to hospitalisation. Future investigations can benefit from more robust research designs, utilising episode-driven data sources that enable a thorough exploration of these connections.

The results of this review have significant implications for clinical practice, healthcare policy, and future research aimed at reducing complications associated with cancer treatment and lowering hospitalisation rates. The findings offer valuable insights and potential strategies, including:

1. Highlighting the need for healthcare reforms and improvements in several countries due to the high rates of hospitalisation resulting from treatment-related complications of cancer.
2. Providing oncologists with a better understanding of the complications associated with therapy-related cancer treatments and their serious repercussions on hospitalisations, enabling them to provide early diagnosis, personalised care plans, and strategies to mitigate the effects.
3. Educating patients and healthcare providers about the potential complications of cancer treatments, facilitating timely identification and intervention, ultimately improving patient outcomes and quality of life.
4. Enabling policymakers to allocate resources more effectively by investing wisely and equitably in cancer preventive strategies, enhancing the quality of care provided to patients.
5. Recommending that healthcare policies incorporate these findings and nations strengthen their preventive measures and care models.
6. Suggesting the need for more research, particularly in low- and lower-middle-income countries, with long-term follow-up studies to assess the impact of treatments. Economically viable interventions and prolonged economic assessments are also recommended.

## Conclusions

This review highlights the increasing burden of post-therapy complications due to systemic therapy and radiotherapy and the lack of data on cancer therapy-related complications in low-resource settings. Most studies were conducted in high-income countries, raising questions about research in low- and lower-middle-income countries. To reduce hospitalisation due to therapy-related complications, we need to invest in research, improve educational resources, increase screening and surveillance programs, and address misconceptions. Future research should be standardised, patient-centred, and investigate health economics.

### Supplementary Information

Supplementary Material 1.

Supplementary Material 2.

## Data Availability

No datasets were generated or analysed during the current study.

## Abbreviations

HICs: High-income countries
LIMCs: Low- and middle-income countries
LOS: Length of stay
SD: Standard deviation
RCTs: Randomized controlled trials
