# Supplementary material for: Systemic therapy and radiotherapy related complications and subsequent hospitalisation rates: a systematic review
Source: BMC Cancer. 2024 Jul 10;24:826. doi: 10.1186/s12885-024-12560-8 (PMC11238411; doi:10.1186/s12885-024-12560-8)
Supplement: Supplementary file 2 — Supplementary Material 2. [file 12885_2024_12560_MOESM2_ESM.docx]

**Appendix Tables**

**Appendix Table A1. Search terms, by search engines.**

| Search engine | Search terms | Outcomes |
| --- | --- | --- |
| **Scopus** | (("Systemic Therapy" OR "Chemotherapy" OR "Immunotherapy" OR "Targeted Therapy" OR "Radiotherapy") AND ("Complications" OR "Adverse Events" OR "Side Effects" OR "Hematological Toxicities" OR "Gastrointestinal Disturbances" OR "Dermatological Reactions" OR "Neurologic Impairments" OR "Cardiopulmonary Sequelae") AND ("Hospitalisation" OR "Inpatient" OR "Admission") AND ("Cancer" OR "Neoplasm" OR "Malignancy")) AND ( LIMIT-TO ( PUBYEAR,2000) OR LIMIT-TO ( PUBYEAR,2001) OR LIMIT-TO ( PUBYEAR,2002) OR LIMIT-TO ( PUBYEAR,2003) OR LIMIT-TO ( PUBYEAR,2004) OR LIMIT-TO ( PUBYEAR,2005) OR LIMIT-TO ( PUBYEAR,2006) OR LIMIT-TO ( PUBYEAR,2007) OR LIMIT-TO ( PUBYEAR,2008) OR LIMIT-TO ( PUBYEAR,2009) OR LIMIT-TO ( PUBYEAR,2010) OR LIMIT-TO ( PUBYEAR,2011) OR LIMIT-TO ( PUBYEAR,2012) OR LIMIT-TO ( PUBYEAR,2013) OR LIMIT-TO ( PUBYEAR,2014) OR LIMIT-TO ( PUBYEAR,2015) OR LIMIT-TO ( PUBYEAR,2016) OR LIMIT-TO ( PUBYEAR,2017) OR LIMIT-TO ( PUBYEAR,2018) OR LIMIT-TO ( PUBYEAR,2019) OR LIMIT-TO ( PUBYEAR,2020) OR LIMIT-TO ( PUBYEAR,2021) OR LIMIT-TO ( PUBYEAR,2022) OR LIMIT-TO ( PUBYEAR,2023) ) AND ( LIMIT-TO ( LANGUAGE,"English" ) ) AND ( LIMIT-TO ( DOCTYPE,"ar" ) ) | 1052 articles |
| **PubMed** | Search: (("Systemic Therapy" OR "Chemotherapy" OR "Immunotherapy" OR "Targeted Therapy") AND ("Radiotherapy" OR "Radiation Therapy") AND ("Complications" OR "Adverse Events" OR "Side Effects" OR "Hematological Toxicities" OR "Gastrointestinal Disturbances" OR "Dermatological Reactions" OR "Neurologic Impairments" OR "Cardiopulmonary Sequelae") AND ("Hospitalisation" OR "Inpatient" OR "Admission") AND ("Cancer" OR "Neoplasm" OR "Malignancy") ) Filters: from 2000/1/1 - 2023/8/30 | 1527 articles |
| **Embase** | ('Systemic Therapy' OR 'Chemotherapy' OR 'Immunotherapy' OR 'Targeted Therapy') AND ('Radiotherapy' OR 'Radiation Therapy') AND ('Complications' OR 'Adverse Events' OR 'Side Effects' OR ‘Hematological Toxicities’ OR ‘Gastrointestinal Disturbances’ OR ‘Dermatological Reactions’ OR ‘Neurologic Impairments’ OR ‘Cardiopulmonary Sequelae’) AND ('Hospitalisation' OR 'Inpatient' OR 'Admission') AND ('Cancer' OR 'Neoplasm' OR 'Malignancy') | 245 articles |
| **Web of Science** | TS=("Systemic Therapy" OR "Chemotherapy" OR "Immunotherapy" OR "Targeted Therapy") AND TS=("Radiotherapy" OR "Radiation Therapy") AND TS=("Complications" OR "Adverse Events" OR "Side Effects" OR “Hematological Toxicities” OR “Gastrointestinal Disturbances” OR “Dermatological Reactions” OR “Neurologic Impairments” OR “Cardiopulmonary Sequelae”) AND TS=("Hospitalisation" OR "Inpatient" OR "Admission") AND TS=("Cancer" OR "Neoplasm" OR "Malignancy") | 137 articles |

**Appendix Table A2. Quality score assessment for cohort study**

| Studies | Quality assessment indicators | | | | | | | | | | | Overall score | Overall appraisal |
| --- | --- | --- | --- | --- | --- | --- | --- | --- | --- | --- | --- | --- | --- |
|  | Q_1_ | Q_2_ | Q_3_ | Q_4_ | Q_5_ | Q_6_ | Q_7_ | Q_8_ | Q_9_ | Q_10_ | Q_11_ |  |  |
| Vangelov et al. (2017)[30] | ✓ | ✓ | ✓ | ✓ | ✓ | ✓ | ✓ | ✓ | ✓ | ✓ | ✓ | 11 | Included |
| Goldstein et al. (2010)[8] | ✓ | ✓ | ✓ | ✓ | ✓ | ✓ | ✓ | ✓ | ✓ | ✓ | ✓ | 11 | Included |
| Ong et al. (2016)[22] | ✓ | ✓ | ✓ | ✓ | ✓ | ✓ | ✓ | ✓ | ✓ | ® | ✓ | 10.5 | Included |
| Hassett et al. (2006)[31] | ✓ | ✓ | ✓ | ✓ | ✓ | ✓ | ✓ | ✓ | ✓ | ✕ | ✓ | 10 | Included |
| O'Neil et al. (2016)[32] | ✓ | ✓ | ✓ | ✓ | ✓ | ✓ | ✓ | ✓ | ✓ | ✕ | ✓ | 10 | Included |
| Schilling et al. (2011)[10] | ✓ | ✓ | ✓ | ✓ | ✓ | ✓ | ✓ | ✓ | ✓ | ✕ | ✓ | 10 | Included |
| Jairam et al. (2019)[9] | ✓ | ✓ | ✓ | ✓ | ✓ | ✓ | ✓ | ✓ | ✓ | ✕ | ✓ | 10 | Included |
| Marvelde et al. (2020)[38] | ✓ | ✓ | ✓ | ✓ | ✓ | ✓ | ✓ | ✓ | ® | ® | ✓ | 10 | Included |
| Alamgeer et al. (2022)[35] | ✓ | ✓ | ✓ | ✓ | ✓ | ✓ | ✓ | ✓ | ✓ | ✓ | ✓ | 11 | Included |
| David et al. (2022)[36] | ✓ | ✓ | ✓ | ✓ | ✓ | ✓ | ✓ | ✓ | ✓ | ✓ | ✓ | 11 | Included |
| Bassal et al. (2021)[37] | ✓ | ✓ | ✓ | ✓ | ✓ | ✓ | ✓ | ✓ | ✓ | ✓ | ✓ | 11 | Included |
| Wijetunga et al. (2021)[33] | ✓ | ✓ | ✓ | ✓ | ✓ | ✓ | ✓ | ✓ | ✓ | ✓ | ✓ | 11 | Included |
| Lang et al. (2008)[45] | ✓ | ✓ | ✓ | ✓ | ✓ | ✓ | ✓ | ✓ | ✓ | ✓ | ✓ | 11 | Included |
| Waddle et al. (2015)[19] | ✓ | ✓ | ✓ | ✓ | ✓ | ✓ | ✓ | ✓ | ✓ | ✕ | ✓ | 10 | Included |
| Fruh et al. (2013)[44] | ✓ | ✓ | ✓ | ✓ | ✓ | ✓ | ✓ | ✓ | ✓ | ® | ✓ | 10.5 | Included |
| Du et al. (2005)[16] | ✓ | ✓ | ✓ | ✓ | ✓ | ✓ | ✓ | ✓ | ✓ | ✕ | ✓ | 10 | Included |
| Ling et al. (2014)[7] | ✓ | ✓ | ✓ | ✓ | ✓ | ✓ | ✓ | ✓ | ✓ | ® | ✓ | 10.5 | Included |
| Ernight et al. (2015)[42] | ✓ | ✓ | ✓ | ✓ | ✓ | ✓ | ✓ | ✓ | ✓ | ® | ✓ | 10.5 | Included |
| Hassett et al. (2011)[40] | ✓ | ✓ | ✓ | ✓ | ✓ | ✓ | ✓ | ✓ | ® | ✕ | ✓ | 9.5 | Included |
| Saxena et al. (2021)[39] | ✓ | ✓ | ✓ | ✓ | ✓ | ✓ | ✓ | ✓ | ® | ✕ | ✓ | 9.5 | Included |
| Du et al. (2002)[47] | ✓ | ✓ | ✓ | ✓ | ✓ | ✓ | ✓ | ✓ | ✓ | ✕ | ✓ | 10 | Included |

Note: ✓ = yes ( = 1), ✕ = no ( = 0), ® = unclear ( = 0.5); quality assessment decision rules for 11 scales [(i) poor if the overall score ≤ 5, (ii) medium if overall scores is 6 to 9, and (iii) high if the overall score>9], Q1. Were the two groups similar and recruited from the same population? Q2. Were the exposures measured similarly to assign people to both exposed and unexposed groups? Q3. Was the exposure measured in a valid and reliable way? Q4. Were confounding factors identified? Q5. Were strategies to deal with confounding factors stated? Q6. Were the groups/participants free of the outcome at the start of the study (or at the moment of exposure)? Q7. Were the outcomes measured in a valid and reliable way? Q8. Was the follow-up time reported and sufficient to be long enough for outcomes to occur? Q9. Was follow-up complete, and if not, were the reasons to loss to follow-up described and explored? Q10. Were strategies to address incomplete follow-up utilized? Q11. Was appropriate statistical analysis used?

**Appendix Table A3. Quality score assessment for randomised control trials**

| Quality assessment indicators | Studies | | |
| --- | --- | --- | --- |
|  | **Gridelli et al. (2012)**[41] | **Hanna et al. (2004)**[43] | **Sederholm et al. (2005)**[46] |
| Question 1: Was true randomization used for assignment of participants to treatment groups? | ✓ | ✓ | ✓ |
| Question 2: Was allocation to groups concealed? | ✓ | ✓ | ✓ |
| Question 3: Were treatment groups similar at the baseline? | ✓ | ✓ | ✓ |
| Question 4: Were participants blind to treatment assignment? | ✓ | ® | ✓ |
| Question 5: Were those delivering the treatment blind to treatment assignment? | ✓ | ✓ | ✓ |
| Question 6: Were treatment groups treated identically other than the intervention of interest? | ✓ | ✓ | ✓ |
| Question 7: Were outcome assessors blind to treatment assignment? | ✓ | ✓ | ✓ |
| Question 8: Were outcomes measured in the same way for treatment groups? | ✓ | ✓ | ✓ |
| Question 9: Were outcomes measured in a reliable way? | ✓ | ✓ | ✓ |
| Question 10: Was follow-up complete and, if not, were differences between groups in terms of their follow-up adequately described and analyzed? | ✓ | ✓ | ✓ |
| Question 11: Were participants analyzed in the groups to which they were randomized? | ✓ | ✓ | ✓ |
| Question 12: Was appropriate statistical analysis used? | ✓ | ✓ | ✓ |
| Question 13: Was the trial design appropriate and any deviations from the standard RCT design (individual randomization, parallel groups) accounted for in the conduct and analysis of the trial? | ✓ | ✓ | ✓ |
| Overall score | 13 | 12.5 | 13 |
| Overall appraisal | Included | Included | Included |

Note: ✓ = yes ( = 1), ✕ = no ( = 0), ® = unclear ( = 0.5); quality assessment decision rules for 13 scales [(i) poor if the overall score ≤ 6, (ii) medium if overall scores is 7 to 10, and (iii) high if the overall score>10],

**Appendix Table A4. Quality score assessment for cross-sectional study**

| Quality assessment indicators | Rivera et al. (2017)[34] |
| --- | --- |
| Q1. Were the criteria for inclusion in the sample clearly defined? | ✓ |
| Q2. Were the study subjects and the setting described in detail? | ✓ |
| Q3. Was the exposure measured in a valid and reliable way? | ✓ |
| Q4. Were objective, standard criteria used for measurement of the condition? | ✓ |
| Q5. Were confounding factors identified? | ✓ |
| Q6. Were strategies to deal with confounding factors stated? | ✓ |
| Q7. Were the outcomes measured in a valid and reliable way? | ✓ |
| Q8. Was appropriate statistical analysis used? | ✓ |
| Overall score | 8 |
| Overall appraisal | Included |

Note ✓= yes ( = 1), ✕ = no ( = 0), ® = unclear (= 0.5); quality assessment decision rules for 8 scales: (i) poor if the overall score <5, (ii) medium if the overall score is 5 to 6, and (iii) high if the overall score > 6
